# Oral Flucloxacillin for Treating Osteomyelitis: A Narrative Review of Clinical Practice

**DOI:** 10.7150/jbji.40667

**Published:** 2020-01-01

**Authors:** Helga Preiss, Philipp Kriechling, Giulia Montrasio, Tanja Huber, İmke Janssen, Andreea Moldovan, Benjamin A. Lipsky, İlker Uçkay

**Affiliations:** 1 Internal Medicine, Baden Hospital, Baden, Switzerland; 2 Orthopaedic Surgery, Balgrist University Hospital, Zurich, Switzerland; 3 Hospital Pharmacy, Balgrist University Hospital, Zurich, Switzerland; 4 Internal Medicine, Zollikerberg Hospital, Zollikon, Switzerland; 5 Infectiology, St. Constantin Hospital, Braşov, Romania; 6 Department of Medicine, University of Washington, Seattle, Washington, USA; 7 Infectiology, Balgrist University Hospital, Zurich, Switzerland

**Keywords:** oral flucloxacillin, osteomyelitis, literature review, clinical treatment, clinical remission

## Abstract

Flucloxacillin (FLU) administered by the oral route is widely used for treating various infections, but there are no published retrospective or prospective trials of its efficacy, or its advantages or disadvantages compared to parenteral treatment or other antibiotics for treating osteomyelitis. Based on published *in vitro* data and expert opinions, other non-β-lactam oral antibiotics that have better bone penetration are generally preferred over oral FLU. We reviewed the literature for studies of oral FLU as therapy of osteomyelitis (OM), stratified by acute versus chronic and pediatric versus adult cases. In striking contrast to the prevailing opinions and the few descriptive data available, we found that treatment of OM with oral FLU does not appear to be associated with more clinical failures compared to other oral antibiotic agents. Because of its narrow antibiotic spectrum, infrequent severe adverse effects, and low cost, oral FLU is widely used in clinical practice. We therefore call for investigators to conduct prospective trials investigating the effectiveness and potential advantages of oral FLU for treating OM.

## Introduction

The traditional antibiotic regimen for a patient who has undergone surgical debridement for osteomyelitis (OM) includes 2-6 weeks of parenterally administered agent(s), followed by an oral course of several weeks 1,2. Parenteral (usually intravenous) antibiotic therapy is associated with several substantial problems: it requires either prolonged hospitalization or treatment by an outpatient parenteral antibiotic therapy (OPAT) unit; the required intravenous catheter (usually a central line) is associated with frequent adverse effects (including infection and thrombosis); and, it is associated with high financial costs. For example, an evaluation by Gardiol et al. 3 of their OPAT program in Switzerland found that 16 of their 179 (9 %) OPAT patients had complications associated with the treatment. The most common were drug-related adverse events, which occurred in ten patients (5.5 %) and required readmission to the hospital in three cases. There were also six line-related (all peripheral) adverse events (3.5 %) 3.

Flucloxacillin (FLU) is a narrow-spectrum, semisynthetic penicillin available in both parenteral and oral formulations 4-8. It is active against aerobic gram-positive pathogens, notably including methicillin-susceptible *Staphylococcus aureus* (MSSA) 8. FLU is closely related to other semisynthetic penicillins (nafcillin, oxacillin, (di)cloxacillin) that have been widely used for decades for a variety of orthopedic and community-acquired skin infections worldwide 8,9. Treatment with FLU has several theoretical advantages over other antibiotic agents for these infections: it has bactericidal properties (potentially important for selected infections); its antibiotic spectrum is limited (reducing risk of antibiotic resistance); it is inexpensive (reducing financial costs); and the two formulations afford the possibility of an early switch from intravenous to oral dosing (reducing risks and potentially allowing earlier hospital discharge) 6. One potentially challenging issue is that the bone penetration of oral β-lactam antibiotics, at least *in vitro,* is poor 4-7. Unfortunately, there are almost no published clinical data on the effectiveness or safely of treatment with oral FLU for osteomyelitis (OM).

This lack of evidence of the usefulness of oral semi-synthetic antibiotic agents has led to international expert groups and guidelines, such as the Infectious Diseases Society of America (IDSA) guidelines 9 and the European Society for Pediatric Infectious Diseases (ESPID) 10 to recommend that the preferred route of administration for treating OM are intravenous FLU or a first-generation cephalosporin 9,10. Nevertheless, clinicians in some countries with a low endemicity of methicillin-resistant *S. aureus* isolates, e.g., the Nordic countries, Baltic States and Netherlands, use oral FLU, at least for the maintenance therapy, of staphylococcal OM 6. Several recent studies have demonstrated that oral β-lactam antibiotics, e.g. co-amoxiclav, can be safely used in many types of bone and joint infections, including for diabetic foot OM 11. In the United Kingdom, the prescribing of oral FLU has increased by 21% during the past decade 12, and oral FLU can be used for OM 13. Considering the potential benefits, and lack of published data, we present this narrative review to address the potential place of oral FLU for treating OM.

## Methods

We performed a narrative literature review by searching for articles in PubMed (using the MeSH terms “oral flucloxacillin”, “*Staphylococcus aureus*” and “osteomyelitis”), Google Scholar and references cited in retrieved articles. We reviewed articles written in English; with correspondence in German, French, Romanian and Turkish languages. Our review only sought papers concerned with oral FLU for treating MSSA infections, not those on intravenous formulations or for treatment of other bacteria. For our inclusion criteria we defined that the duration of oral FLU used in the study must be more than the half of the total antibiotic course. We did not consider antibiotic dosing of oral FLU considerations or minimal clinical follow-up time of enrolled patients as inclusion criteria. We excluded studies: on animals; using combination therapies with oral rifampin; reporting on infected implants or soft tissue infections; on virulence analyses 14; on *S. aureus* body carriage or colonization 15; on pharmacokinetic-pharmacodynamics (PK/PD) issues 16; or, on minimal inhibitory concentration (MIC) assessments 17,18. We also excluded papers predominatingly related to only related antibiotic regimens, such as nafcillin, oxacillin, or (di)-cloxacillin. Additionally, we explored data on the levels of oral FLU use in Switzerland during the past decade.

## Results

### General results of the literature search

**Figure 1:**
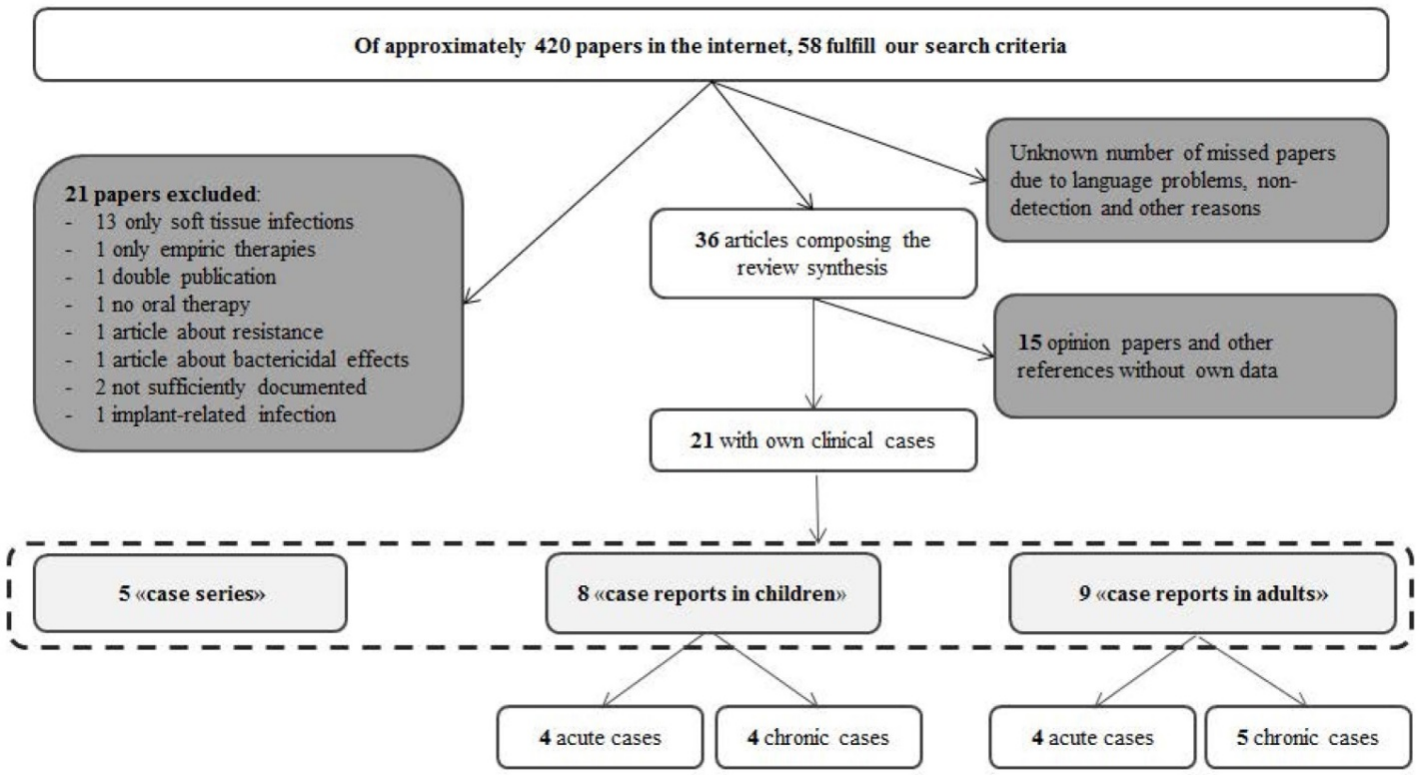
Review flowchart

Our review produced 420 articles, only 58 of which met our criteria and we used these to synthetize our review. Papers with own clinical cases of oral FLU use in OM are analyzed more in detail (Figure 1). We stratified them as involving childhood or adult OM cases, acute or chronic OM cases, and between case reports and case series (Tables 1-5). Although many of the included papers provided information on the feasibility of oral FLU therapy for OM, this was not the underlying primary issue of any. As the heterogeneity and the large amount of missing information in the papers were so great, we could not attempt any type of meta-analyses of our results. Of note, most of the studies were conducted in English speaking (particularly the United Kingdom) and Nordic countries.

### Authority recommendations for prescription of oral flucloxacillin in Switzerland

According to the most frequently used Swiss Federal documentation 40, general indications for treatment with FLU are the same as in most other countries: skin and soft tissue infections, infection with MSSA, susceptible coagulase-negative staphylococci, as well as streptococci and other non-staphylococcal gram-positive bacteria (*albeit as a weak recommendation*). Importantly, they do not specify if the route of administration routes should be different for bone than for soft tissue infections. Among Swiss Infectious Diseases physicians, treating with oral FLU for OM is unpopular for the treatment of OM (*personal communications*). One example of a related consequence is that in Switzerland, because prospective trials involving use of oral antibiotic therapy must pass through the Ethical Committees, researchers avoid using oral FLU as a therapeutic option for studies of bone and joint infections 41. This is not the same in other countries 42. For example, in Sweden, Fiji and Australia, oral FLU can be officially used for studies of treatment of OM 43-46.

### Official prices and registered use of oral flucloxacillin in Switzerland

The daily price for treatment with oral FLU (500 mg, 3-4 times a day) on the Swiss market is five Swiss Francs ($5 US dollars in October 2019). The quantity of oral FLU prescribed in Switzerland between 2004 and 2017 was 0.13 DDD (defined daily dose) per 1000 patient-months. There was a two-fold increase in use during this time period in the French-speaking part of the country, a 29% increase in the Italian-speaking part, and an 18% in the German-speaking majority regions (ANRESIS databases; 47). In our Balgrist University Hospital, a referral center for orthopedic infections, the corresponding DDD was 0.72 per 1000 patient-months in 2017. In comparison, in the United Kingdom (the country with the most publications regarding oral FLU in OM), the average monthly prescription rates increased from 4.74 per 1000 patient-months in 2004 to 5.74 in 2013, an increase of 21.1% 12. We do not know the reasons for this increase in prescribing of oral FLU, but it might be that British physicians chose to prescribe more of the 500 mg tablets the 250 mg capsules 12. In Sweden, the usual daily dosing of oral FLU is 1.5 g three times daily for OM and septic arthritis 44. The EUCAST Clinical Breakpoint Tables (European dosing recommendations) promote a “standard dose” of oral FLU of 1.0 g three times daily; and a “high oral dose” of 1g four times daily in some cases 48.

### International guidelines

To our knowledge, treatment with oral FLU is not a primary choice for OM in any internationally accepted guidelines. For example, the ESPID guidelines 10 recommend initial therapy with a 1^st^ or 2^nd^ generation cephalosporin (for other susceptible bacteria other than MSSA). For treating adult in the OM population, the IDSA guidelines 9 advocate 1.5-2.0 g of intravenous oxacillin (the nearest equivalent to flucloxacillin) every four hours. Both guidelines consider almost all other oral β-lactams, as a group and independently of their dosing, inappropriate due to poor bone penetration *in vitro* (with the possible exception or amoxicillin and co-amoxiclav in certain circumstances); this is true even when the patient has shown a good response to initial parenteral therapy treatment with a non-preferred agent. In striking contrast, after a favorable clinical response to parenteral treatment, guidelines encourage switching to oral antibiotic classes for almost all other agents (e.g., quinolones, linezolid, clindamycin, co-trimoxazole, tetracyclines, and metronidazole) provided there is 9. The failure to recommend oral FLU also applies for DFO in the US guidelines 49.

### Regional and national recommendations

In contrast to their international counterparts, many national guidelines explicitly recommend treatment with oral FLU for MSSA OM*,* generally citing the favorable personal experience of the author groups 12. A UK study reported that general practitioners treated 394 OM episodes at least partially with oral FLU, with generally favorable results 12. Recent recommendations from South Korea also mention the possibility of post-surgical treatment of OM with oral FLU 50. Swedish 44, Australian 46 and Fijian 45 guidelines also support treatment with oral FLU in OM, including spondylodiscitis 44. Similarly, the national Australian Antibiotic Guidelines (*Therapeutic Guidelines*) promote oral FLU as a first line treatment for acute and chronic OM, albeit following a 2-4 week course of intravenous FLU 46.

### Published clinical efficacy of oral flucloxacillin in osteomyelitis

Tables 1-5 present summaries of the published clinical literature on treatment of OM with oral FLU. Over twenty years ago Lowden et al. reported that among five children with clavicular OM 19 four (80%) relapsed after three weeks intravenous FLU followed by three to four weeks of oral FLU. The author`s attributed this high recurrence rate to the clavicle being a difficult anatomic site to treat. No subsequent studies have reported such a high failure rate, suggesting there may have been other problems in the management of these patients 19. Vinod et al. reported their experience with children treated for OM or septic arthritis 20. In 77% of the cases the children received intravenous FLU followed by oral FLU. Overall, the recurrence rate was only 1.4%, demonstrating the efficacy of oral FLU in treating hematogenous OM 20. Similarly, Nunn et al. demonstrated efficacy in the majority (exact numbers are not provided) of 43 evaluable children treated in an African hospital for OM with oral FLU following initial intravenous therapy 51. In 2001 Beronius et al. published their experience in Sweden of treating 42 episodes of spondylodiscitis for a median of 10 days with intravenous antibiotics (cefuroxime in two-thirds of cases), followed by a median of 179 days of oral FLU, none of which were associated with a relapse 23. For the subset of patients with DFO, we found no well-designed trials comparing oral FLU with other antibiotic regimens. One study specifically comparing patients treated with oral β-lactam antibiotics to those treated with oral quinolones for DFO found no significant difference in the remission rates 52. Several case series, reports and surveys have demonstrated the feasibility of treatment with oral β-lactam therapy in DFO 3,36. Interestingly, a survey of Australasian infectious diseases clinicians found that oral FLU is the maintenance antibiotic treatment of choice, associated with good clinical responses, for staphylococcal DFO 53.

In sum, the experience of the majority publications reporting on outcomes of treatment with oral FLU for OM have demonstrated good efficacy. While these studies used different dosing regimens and treatment durations, and had non-uniform rates of surgical treatment (Tables 1-5), many used oral FLU as a maintenance therapy after a course of initial parenteral antibiotic therapy. Among the reported cases series there were relatively few documented failures (Tables 1, 2 and 4). In one case, the authors treated a pediatric spondylodiscitis with oral FLU 27 and incriminate the relatively short duration of therapy (24 days), not the choice of FLU, as the cause of relapse. Other reported recurrences occurred in studies from New Zealand 31 and Denmark 36.

### Drug interactions and tolerability of oral flucloxacillin in the treatment of osteomyelitis

Although comparative large epidemiological data are lacking, according to personal experience of various authors (*personal communications*) and published literature, oral FLU probably causes not more adverse events than other penicillins. The most common reported side effects include hypersensitivity, gastrointestinal symptoms, exanthema, nephrotoxicity, myelotoxicity, oxoproline acidosis. Drug-drug interactions have been reported with paracetamol (acetaminophen) 40,42, and rarely with warfarin is 54 and rifampin. In a study in 15 adult patients with various osteoarticular infections 55 the combined treatment with intravenous FLU and oral rifampicin significantly increased serum FLU levels by 45% in 10 patients, while a decrease was observed in one patient. The clinical significance of this finding remained unknown, since all infections were cured 55. It is likely that oral FLU could also be associated with such an interaction, although possibly at a subclinical level. Contrary to common belief, it is probably not necessary to administer oral FLU on an empty stomach, as concomitant food intake does not appear to alter the pharmacokinetic parameters 56. One other interaction of note is that combining treated with FLU (like other penicillins) with probenecid commonly can boost the serum FLU concentrations 8,40,42.

## Discussion

Our review of the literature revealed that despite its widespread use around the world, there is remarkably little documentation of the clinical experience of treatment with oral FLU alone for the treatment of acute or chronic, implant-free, OM in children or adults. We found no randomized trials or retrospective multivariate analyses that adjusted for the large case-mix of included subjects.

The few published reports of case series clearly reflect the personal experience of the authors, and are almost exclusively composed of case reports and small case series. The information on efficacy and adverse events if present at all, is largely hidden within the reports, as the primary objectives of the studies differed from those of our review.

Based on *in vitro* data, treatment with oral FLU (as with all oral β-lactam agents) warrants careful observation when used for treating OM. In most studies the ratios of bone to intravenous concentrations for β-lactams are about 5-20%, with a very wide inter-individual variation in the availability of oral FLU. As an example, the serum trough levels reported by Gath et al. were 15±5.9 mg/L 5, while the peak levels reported by Dijkmans et al were 22-26 mg/L (range, 7-53 mg/L) 6. Thus, while most intravenous (and probably intramuscular) β-lactams reach the necessary MIC for MSSA in bone 57-61, this might not be the case with oral administration. One cause for the relatively low levels achieved with oral FLU is likely the innate variabilities in its gastrointestinal absorption 6. Among humans, 9% largely fail to absorb FLU and another 17% are poor absorbers 61. A pharmacologic *in vivo* study from the Netherlands 61 compared levels of cefuroxime and FLU when given by oral versus parenteral routes in plasma and intraoperative bone during twenty hip and knee arthroplasty surgeries. The authors allocated patients to four groups: either 1 x 500 mg or 7 x 500 mg oral cefuroxime, following 2000 mg intravenous FLU; and either 1 x 500 mg or 7 x 500 mg oral FLU followed by 1500 mg parenteral cefuroxime. All oral administrations failed to achieve a measurable osseous concentration 60. FLU is a highly protein bound drug and it is predominantly the free drug that is active in the killing of bacteria. Even with a relatively high dosage of 1 g q.i.d, free plasma drug concentrations might drop below the MIC of a typical strain of MSSA (0.25 mg/l) at 50% of the dosing interval 56. Given a drug penetration into bone ratio of 10-20%, it is unlikely that a given dose of oral FLU will achieve therapeutic concentrations in the bone for several hours. We only found one published study reporting the contrary: Köndell et al. found a mandibular bone concentration of 3.8 mg/L after oral administration of FLU, which would likely be therapeutically adequate for treating MSSA OM 62.

A key issue is that serum antibiotic concentration measurements do not reflect the entire pharmacologic picture, as it is not a highly predictable surrogate for the actual concentration of the administered antibiotic in infected bone 16. Likewise, information is lacking regarding the relationship between serum antibiotic concentrations over time and antibiotic effectiveness (time-over minimal inhibitory concentration [MIC]) for FLU and MSSA), as FLU is not an antibiotic that is peak concentration-dependent (as is true of all β-lactam antibiotics agents). Interested readers can review a paper in which members of our group recently summarized insights on the pharmacokinetic-pharmacodynamics of antibiotics in the bone 16. The effectiveness of antibiotic treatment for OM is complex, depending on the interaction of drug, host, microbial agent and other aspects. For example, it is disturbing that Alvarez-Ferrero et al. 61 failed to detect a measurable osseous concentration *in vivo* after administration of oral FLU, but they did not address the large technical and analytical problems in assessing drug concentrations in bone 61. There are likely also differences in antibiotic concentrations in cancellous versus cortical versus mixed homogenized bone, in infected versus non-infected bone, and issues related to blood contamination during bone sampling. Therapeutic drug monitoring (TDM) might be a solution to concerns about therapeutic concentration. In patients being treated with FLU use (oral or IV), several centers have introduced such TDM policies, but its clinical benefit remains unproven. Most TDM publications to date provide only laboratory (not clinical) data for FLU 63. This contrasts to antibiotics such as cefepime, for which TDM has been shown to help prevent potentially severe adverse events 64.

The outcome of oral FLU treatment of OM does not appear to be worse than with other oral drugs 2, although direct comparative data are lacking. So, why does treatment with oral FLU seem to be effective for OM if its bone penetration appears to be limited? In our review (Tables 1-5), we found few reported failures of OM treatment, either in adults or children. Likewise, in the study by Vinod et al., of 32 children with OM treated with IV FLU for a median of three days, followed by oral therapy for a median of 6 weeks, the relapse rate was only 1.4% 20. We can only speculate on the reasons. Based on our review and *personal communications*, even in “*oral FLU-supporting countries*” 44-46, treatment of acute as well as chronic OM is almost always started with intravenous FLU for about two weeks, which is then followed by a longer period of oral FLU. Official guidelines in Australia 43,46 have been recommending a much longer intravenous duration for OM compared to what is currently in place in Switzerland (~ 2 weeks or less). It is possible that long oral maintenance antibiotic therapy is unnecessary, as an unimpaired immune system is able to eradicate the remaining bacteria 1,41, including for DFO 44,45,52. This is why in this review we elected to circumvent the confounding bias of long duration intravenous therapy by targeted OM episodes in which ≥ 50% of the course was with oral FLU.

There is more data supporting the clinical efficacy in treating OM in various settings with oral β-lactams other than FLU. For example, many experts in the Nordic countries consider cloxacillin (which is not FLU, *sensu strictu*) the preferred oral β-lactam drug. For example, Bachur et al. found good outcomes with a four-day intravenous administration of cefazolin with an early switch to oral cephalexin for 28 days in stable OM 65. Similarly, Le Saux found no superiority for a long-duration intravenous β-lactam administration in children with hematogenous OM 66 compared to a short duration. Likewise, Zaoutis et al. in a retrospective study of hematogenous pediatric OM, treatment with prolonged intravenous β-lactam therapy compared to an early switch to oral therapy gave similar outcomes at six months 67. Several pediatric studies in the 1970s and 1980s summarized the feasibility of oral β-lactam care for OM 11. The recently published OVIVA trial prospectively randomized various types of sever bone and joint infections, including infected arthroplasties and osteomyelitis, into either six weeks of intravenous antibiotic therapy or a switch to oral antimicrobials after up to 7-days of initial parenteral therapy 7. Roughly 38% of all participants had infections with MSSA. Overall, 74 patients in the oral arm were treated with oral penicillins and clinical failure was noted in 17 (23%), compared to 9 of 57 (16%) in the intravenous arm, a non-significant difference (7; Supplementary Appendix).

There is also a great variability in antibiotics used in patients with DFO, with co-amoxiclav among the most frequently orally administered β-lactams worldwide 11. In a retrospective cohort analysis of a clinical pathway for patients with DFO, the authors conducted a cluster-controlled Cox regression model with an emphasis on the more than 300 patients treated with oral co-amoxiclav (at a median dose of 2000 mg BID/day) 11. After a median follow-up of three years, DFO recurred in 22% of the patients, which is compatible with rates reported for various treatments in the literature. In multivariate analysis, treatment with oral co-amoxiclav did not influence the likelihood of remission or recurrence, either when used as the only agent from the start, or when administered for >50% of the entire antibiotic course 11. Likewise, in a prospective trial by Lázaro-Martínez et al. randomizing patients with DFO to either primarily antibiotic treatment (24 cases with oral co-amoxiclav and 24 cases with other oral antimicrobials) for ninety days or to “conservative” surgery and antibiotics for 10 days 68, there were no significant differences in outcomes. In contrast to the situation with oral FLU, some international guidelines 49 suggest co-amoxiclav is a viable oral antibiotic option for treating DFO 11. These data from studies of other oral β-lactam agents also support the use of oral FLU as a possible alternative in OM.

Our narrative review has several limitations, the most important of which is the heterogeneity of practice regarding treatment with oral FLU. Considering that in some countries oral FLU use is already firmly established and recommended in national guidelines 44-46, there are probably tens of thousands of OM cases treated each year in this way. Unfortunately, these clinicians have rarely published their experience. For this review we specifically avoided contacting these colleagues, in order to base our conclusions on internationally published reports and not just *personal communications*. One alternative would have been to send questionnaires to treating clinicians around the world. Such as task is time-consuming, difficult and prone to selection biases; we know of no exhaustive list of infectious diseases experts in bone and joint infections. There is a Philadelphia Consensus 69 group that probably comes closest to what we imagine of such a community, but it is also composed of invited persons identified by their academic activity; without representing an exhaustive list of experts.

## Conclusions

Based only on the limited *in vitro* data for penetrating into bone, oral FLU could be seen as inferior to other oral agents that are not β-lactams, for which a bone penetration may be better. However, the role of antibiotic penetration, as commonly measured, in outcomes of treatment of bone infection is far from clear 70. Furthermore, most infectious diseases experts lack extensive personal experience in administering oral FLU as treatment for OM, while the small number who do (mostly from English-speaking or Northern European countries) have apparently not seen the need to publish their experience. The few published clinical studies clearly demonstrate that after any necessary debridement, a short course of intravenous FLU followed by oral FLU is a safe and effective option for treating OM (perhaps including DFO) in adults and children.

We encourage those who treat OM patients to consider conducting prospective trials, as treatment with oral FLU has several important advantages to make it worth investigating in in light of the problem of increasing antibiotic resistance: relatively low risk for adverse events and drug interactions; a narrow antibiotic spectrum; and, relatively low costs. Optimally, these studies would be randomized controlled trials comparing oral FLU to an oral quinolone. Statistically, the first option would probably require 2 x 232 OM episodes in a standard binary outcome non-inferiority trial with inferiority limits of 15%. Alternatively, even a prospective cohort study with oral FLU would be useful.

## Figures and Tables

**Table 1:** Case series in humans of chronic osteomyelitis

Reference, Year (Country)	Patients, Age	IV treatment	Duration of oral FLU	Follow-up	Outcome
Lowden et al, 1997 (New Zealand)^19^	5 patients 7-8 years	60% drainage 21 days FLU IV	21-42 days	0.5-1 year	80% relapse recovery not documented
Vinod et al, 2001 (United Kingdom)^20^	32 patients; 3 months to 16 years	49% drainage IV therapy 3 days	21-42 days	66% > 12 months 34% < 12 months	relapse rate 1.4%
Sur et al, 2015 (United Kingdom)^21^	55 patients 26-96 years	30% debridement 40% FLU IV, 24% vancomycin	not known	not known	not known
Alonge et al, 2002 (Nigeria)^22^	3 patients	not known	6 weeks	not known	no relapse
Beronius et al, 2001 (Sweden)^23^	42 patients	median 10 days	179 days	not known	no relapse

FLU = Flucloxacillin; IV = intravenous.

**Table 2:** Case reports of treatment of children with acute osteomyelitis

Reference, Year (Country)	Pathogenesis	Pathogen	Treatment Surgery, IV antibiotic	Duration of Oral FLU	Outcome
Hughes et al, 2016 (United Kingdom)^24^	Hematogenous	MSSA	No operation FLU IV for 4 weeks	14 days	no relapse
Pabla et al, 2011 (United Kingdom)^25^	Traumatic	Unknown	No operation FLU & Penicillin IV	64 days	no relapse
Chiappini et al, 2016 (Italy)^26^	Microtrauma	MSSA	Drainage 7 days ceftazidime IV	28 days	no relapse
Dahal et al, 2019 (Nepal)^27^	Spondylodiscitis	MSSA	10 days vancomycin IV	14 days	1 relapse

FLU = Flucloxacillin; IV = intravenous; MSSA = Methicillin-susceptible *Staphylococcus aureus.*

**Table 3:** Case reports of treatment of children with chronic osteomyelitis

Study	Pathogenesis	Pathogen	Treatment Surgery, IV antibiotic	Duration of oral FLU	Outcome
El Mezouar et al, 2014 (Morocco)^28^	Trauma	MSSA	No operation FLU IV for 6 weeks	120 days	no relapse
Freeman et al, 2008 (Malawi)^29^	Trauma	No culture	Sequestrectomy; fibula transfer bone filling with gentamicin & vancomycin	not known	residual handicap no relapse
Rajakulendran et al, 2013 (United Kingdom)^30^	PIN-tract infection	MSSA	Not known	85 days	no relapse
Kerr et al, 2019 (United Kingdom)^13^	Acetabular osteomyelitis	MSSA	Not known	28 days	no relapse

FLU = Flucloxacillin; IV = intravenous; MSSA = Methicillin-susceptible *Staphylococcus aureus.*

**Table 4:** Case reports of treatment of adults with acute osteomyelitis

Study	Pathogenesis	Pathogen	IV treatment	Duration of oral FLU	Recovery
Waterhouse et al, 2013 (New Zealand)^31^	Hematogenous	MSSA,* H. parainfluenzae*	6 days Augmentin PO 2 days FLU IV	not known, long	1 relapse
Cheer et al, 2009 (UK)^32^	Microtrauma	MSSA	6 weeks FLU	not known, long	No relapse
Marshman et al, 2008 (UK)^33^	Hematogenous	MSSA	12 days FLU IV	28 days	No relapse
Erturan et al, 2012 (UK)^34^	Hematogenous	*C. pseudodiphteriticium*	Synovectomy, 8 days FLU IV 8 days vancomycin IV	42 days	No relapse

*C. = Corynebacterium*; FLU = Flucloxacillin; IV = intravenous; PO = orally; MSSA = Methicillin-susceptible *Staphylococcus aureus*

**Table 5:** Case reports of treatment of adults with chronic osteomyelitis

Study	Pathogenesis	Pathogen	IV treatment	Duration of oral FLU	Recovery
Opara et al, 2007 (United Kingdom)^35^	Hematogenous	MSSA, TB	2 weeks FLU & Fusidic acid IV	14 days 42 days	Residual handicap, no relapse One relapse
Jeppesen et al, 2015 (Denmark)^36^	Hematogenous	MSSA	No surgery	84 days	No relapse
Lindau et al, 2015 (United Kingdom)^37^	Traumatic	Not known	Gentamicin beads	56 days	No relapse
Torda et al, 1995 (Australia)^38^	Spondylodiscitis	MSSA	Not known	280 days	No relapse
Padala et al, 2013 (UK)^39^	Spondylodiscitis	MSSA	Fusidic acid	90 days	No relapse

FLU = Flucloxacillin; IV = intravenous; MSSA = Methicillin-susceptible *Staphylococcus aureus*; TB = Tuberculosis

## References

[1] Rod-Fleury T, Dunkel N, Assal M (2011). Duration of post-surgical antibiotic therapy for adult chronic osteomyelitis: a single-centre experience. Int Orthop.

[2] Uçkay I, Jugun K, Gamulin A, Wagener J, Hoffmeyer P, Lew D (2012). Chronic osteomyelitis. Curr Infect Dis Rep.

[3] Gardiol C, Voumard R, Cochet C, de Vallière S (2016). Setting up an outpatient parenteral antimicrobial therapy (OPAT) unit in Switzerland: review of the first 18 months of activity. Eur J Clin Microbiol Infect Dis.

[4] Landesdorfer C, Bulitta J, Kinzig M (2009). Penetration of Antibacterials into Bone. Clin Pharmacokinet.

[5] Gath J, Charles B, Sampso J, Smithurs B (1995). Pharmacokinetics and Bioavailability of Flucloxacillin in Elderly Hospitalized Patients. J Clin Pharmacol.

[6] Dijkmans C, Hartigh J den, van Dissel JT, Burggraaf J (2012). A Simplified Oral Flucloxacillin Absorption Test for Patients Requiring Long-Term Treatment.

[7] Li HK, Rombach I, Zambellas R (2019). Oral versus Intravenous Antibiotics for Bone and Joint Infection. N Engl J Med.

[8] Zhou Q, Ruan Z, Yuan H, Jiang B, Xu D (2007). RP-HPLC analysis of flucloxacillin in human plasma: Validation and application to a bioequivalence study.

[9] Berbari EF, Kanj SS, Kowalski TJ (2015). 2015 Infectious Diseases Society of America (IDSA) Clinical Practice Guidelines for the Diagnosis and Treatment of Native Vertebral Osteomyelitis in Adults. Clin Infect Dis.

[10] Saavedra-Lozano J, Falup-Pecurariu O, Faust SN (2017). Bone and joint infections. Ped Infect Dis J.

[11] Gariani K, Lebowitz D, Kressmann B (2019). Oral amoxicillin-clavulanate for treating diabetic foot infections. Diabetes Obes Metab.

[12] Francis NA, Hood K, Lyons R, Butler CC (2016). Understanding flucloxacillin prescribing trends and treatment non-response in UK primary care: A Clinical Practice Research Datalink (CPRD) study. J Antimicrob Chemother.

[13] Kerr BD, Finlayson GD, Thompson LJ, McConway JH (2017). Full resolution of subacute acetabular osteomyelitis in an 11-year-old patient following nonsurgical management. J Orthop Traumatol Rehabil.

[14] Post V, Wahl P, Uçkay I (2014). Phenotypic and genotypic characterisation of *Staphylococcus aureus* causing musculoskeletal infections. Int J Med Microbiol.

[15] Bouvet C, Gjoni S, Zenelaj B, Lipsky BA, Hakko E, Uçkay I (2017). *Staphylococcus aureus* soft tissue infection may increase the risk of subsequent staphylococcal soft tissue infections. Int J Infect Dis.

[16] Deabate L, Pagani L, Uçkay I (2014). Modern Antibiotic Treatment of Chronic Long Bone Infections in Adults-Theory, Evidence and Practice. Mediter J Infect Microbes Antimicrob.

[17] Vaudaux P, Ferry T, Uçkay I (2012). Prevalence of isolates with reduced glycopeptide susceptibility in orthopedic device-related infections due to methicillin-resistant *Staphylococcus aureus*. Eur J Clin Microbiol Infect Dis.

[18] Uçkay I, Bernard L, Buzzi M (2012). High prevalence of isolates with reduced glycopeptide susceptibility in persistent or recurrent bloodstream infections due to methicillin-resistant *Staphylococcus aureus*. Antimicrob Agents Chemother.

[19] Lowden CM, Walsh SJ (1997). Acute staphylococcal osteomyelitis of the clavicle. J Pediatr Orthop.

[20] Vinod MB, Matussek J, Curtis N, Graham HK, Carapetis JR (2002). Duration of antibiotics in children with osteomyelitis and septic arthritis. J Paediatr Child Health.

[21] Sur A, Tsang K, Brown M, Tzerakis N (2015). Management of adult spontaneous spondylodiscitis and its rising incidence. Ann R Coll Surg Engl.

[22] Alonge T, Ogunlade S, Omololu AB, Fashina AL, Oluwatosin A (2002). Management of chronic osteomyelitis in a developing country using ceftriaxone-PMMA beads: An initial study. Int J Clin Pract.

[23] Beronius M, Bergman B, Andersson R (2001). Vertebral osteomyelitis in Göteborg, Sweden: a retrospective study of patients during 1990-95. Scand J Infect Dis.

[24] Hughes L, Pace A (2016). The Unusual Case of Osteomyelitis of the Acromion. Int J Orthop.

[25] Pabla R, Tibrewal S, Ramachandran M, Barry M (2011). Primary subacute osteomyelitis of the talus in children: a case series and review. Acta Orthop Belg.

[26] Chiappini E, Mastrangelo G, Lazzeri S (2016). A case of acute osteomyelitis: An update on diagnosis and treatment.

[27] Dahal RK, Khan JA, Bijukachhe B (2019). Recurrence of spinal epidural abscess after inadequate antibiotic dosage regimen. Grand Med J.

[28] El Mezouar I, Abourazzak FZ, Mansouri S, Harzy T (2014). Septic arthritis of the pubic symphysis: a case report. Pan Afr Med J.

[29] Freeman RT, Harrison WJ (2008). Ward round-a football injury?. Malawi Med J.

[30] Rajakulendran K, Picardo NE, El-Daly I, Hussein R (2016). Brodie's abscess following percutaneous fixation of distal radius fracture in a child. Strat Traum Limb Recon.

[31] Waterhouse D, Hornibrook J (2013). A rare cause of nasal septal abscess. NZ Med J.

[32] Cheer K, Shearman C, Jude EB (2009). Managing complications of the diabetic foot. BMJ.

[33] Marshman LAG, Bhatia CK, Krishna M, Friesem T (2008). Primary erector spinae pyomyositis causing an epidural abscess: case report and literature review. Spine J.

[34] Erturan G, Holme H, Smith R, Dodds R, Iyer S (2012). Successful use of daptomycin in Panton-Valentine leucocidin positive *Staphylococcus aureus* paediatric osteomyelitis. Int J Surg Case Rep.

[35] Opara TN, Gupte CM, Liyanage SH, Poole S, Beverly MC (2007). Tuberculous arthritis of the knee with *Staphylococcus* superinfection. J Bone Joint Surg Br.

[36] Jeppesen SM, Frokjaer J, Yderstraede K (2015). Conservative treatment in a patient with diabetic osteomyelitis: antibiotic treatment is sufficient for complete bone regeneration in selected cases. BMJ Case Rep.

[37] Lindau T, Oestreich K (2015). Brodie's Abscess 7 Years after External Fixation of a Displaced Distal Radius Fracture: A Case Report and Review of the Literature. MOJ Ortho Rheuma.

[38] Torda AJ, Gottlieb T, Bradbury R (1995). Pyogenic Vertebral Osteomyelitis: Analysis of 20 Cases and Review. Clin Infect Dis.

[39] Padala PR, Arafa M, Jackowski A (2003). Cervical epidural abscess and osteomyelitis of C-5 vertebra following percutaneous transluminal coronary angioplasty. SICOT Online Report E051.

[40] www.compendium.ch.

[41] Benkabouche M, Racloz G, Spechbach H, Lipsky BA, Gaspoz JM, Uçkay I (2019). Four versus six weeks of antibiotic therapy for osteoarticular infections after implant removal: a randomized trial. J Antimicrob Chemother.

[42] Menezes MN, de Marco BA, Fiorentino FAM, Zimmermann A, Kogawa AC, Salgado HRN (2019). Flucloxacillin: A Review of Characteristics, Properties and Analytical Methods. Crit Rev Anal Chem.

[43] Australian Product Information - APO - Fluocloxacillin (2019). CAS number 34214-51-2. Date of revision. 29 May 2018.

[44] https://infektion.net/wp-content/uploads/2018/11/2018-vardprogram-led-och-skelettinfektioner-final-2018-11-29.pdf

[45] https://www.health.gov.fj/wp-content/uploads/2018/03/Antibiotic-Guidelines.pdf

[46] https://tgldcdp.tg.org.au/guideLine?guidelinePage=Antibiotic&frompage=etgcomplete

[47] http://www.anresis.ch/index.php/indexen.html

[48] http://www.eucast.org/fileadmin/src/media/PDFs/EUCAST_files/Breakpoint_tables/Dosages_EUCAST_Breakpoint_Tables_v_9.0.pdf

[49] Lipsky BA, Berendt AR, Cornia PB (2012). 2012 Infectious Diseases Society of America Clinical Practice Guideline for the Diagnosis and Treatment of Diabetic Foot Infections. Clin Infect Dis.

[50] Kim BN, Kim ES, Oh MD (2014). Oral antibiotic treatment of staphylococcal bone and joint infections in adults. J Antimicrob Chemother.

[51] Nunn T, Rollinger P (2007). Haematogenous pyogenic bone and joint sepsis - Reducing avoidable morbidity. South African Med J.

[52] Lipsky BA, Baker PD, Landon GC, Fernau R (1997). Antibiotic therapy for diabetic foot infections: comparison of two parenteral-to-oral regimens. Clin Infect Dis.

[53] Commons RJ, Raby E, Athan E (2018). Managing diabetic foot infections: a survey of Australasian infectious diseases clinicians. J Foot Ankle Res.

[54] Merwick Á, Niamh Hannon N, Kelly PJ, O'Rourke K (2010). Warfarin-flucloxacillin interaction presenting as cardioembolic ischemic stroke. Eur J Clin Pharmacol.

[55] Garzoni C, Uçkay I, Belaieff W (2014). *In vivo* interactions of continuous flucloxacillin infusion and high-dose oral rifampicin in the serum of 15 patients with bone and soft tissue infections due to *Staphylococcus aureus* - a methodological and pilot study. Springer Plus.

[56] Gardiner SJ, Drennan PG, Begg R (2018). In healthy volunteers, taking flucloxacillin with food does not compromise effective plasma concentrations in most circumstances. PLoS One.

[57] Unsworth PF, Heatley FW, Phillips I (1978). Flucloxacillin in bone. J Clin Pathol.

[58] Holm S, Larsson SE (1982). The penetration of flucloxacillin into cortical and cancellous bone during arthroplasty of the knee. Int Orthop.

[59] Torkington MS, Davison MJ, Wheelwright EF (2017). Bone penetration of intravenous flucloxacillin and gentamicin as antibiotic prophylaxis during total hip and knee arthroplasty.

[60] Parsons RL, Beavis JP, Laurence M, David JA, Paddock GM, Trounce JR (1978). Plasma, bone, hip capsule and drain fluid concentrations of ampicillin and flucloxacillin during total hip replacement after intravenous bolus injection of Magnapen. Br J Clin Pharmacol.

[61] Alvarez Ferrero MM, Vree TB, Baars AM, Slooff TJ (1993). Plasma and bone concentrations of cefuroxime and flucloxacillin. Oral versus parenteral administration in 20 arthroplasties. Acta Orthop Scand.

[62] Köndell PA, Nord CE, Nordenram A (1982). Concentrations of cloxacillin, dicloxacillin and flucloxacillin in dental alveolar serum and mandibular bone. Int J Oral Surg.

[63] Pinder N, Brenner T, Swoboda S, Weigand MA, Hoppe-Tichy T (2017). Therapeutic drug monitoring of beta-lactam antibiotics - Influence of sample stability on the analysis of piperacillin, meropenem, ceftazidime and flucloxacillin by HPLC-UV. J Pharm Biomed Anal.

[64] Huwyler T, Lenggenhager L, Abbas M (2017). Cefepime plasma concentrations and clinical toxicity: a retrospective cohort study. Clin Microbiol Infect.

[65] Bachur R, Pagon Z (2007). Success of short-course parenteral antibiotic therapy for acute osteomyelitis of childhood. Clin Pediatr (Phila).

[66] Le Saux, N, Howard A, Barrowman NJ, Gaboury I, Sampson M, Moher D (2002). Shorter courses of parenteral antibiotic therapy do not appear to influence response rates for children with acute hematogenous osteomyelitis: a systematic review. BMC Infect Dis.

[67] Zaoutis T, Localio AR Leckerman K, Saddlemire S Bertoch D, Keren R (2009). Prolonged intravenous therapy versus early transition to oral antimicrobial therapy for acute osteomyelitis in children. Pediatrics.

[68] Lázaro-Martínez JL, Aragón-Sánchez J, García-Morales E (2014). Antibiotics versus conservative surgery for treating diabetic foot osteomyelitis: A randomized comparative trial. Diabetes Care.

[69] Gehrke T, Parvizi J (2013). 2013 Proceedings of the International Consensus Meeting on Periprosthetic Joint Infection.

[70] Spellberg B, Lipsky BA (2012). Systemic antibiotic therapy for chronic osteomyelitis in adults. Clin Infect Dis.

